# Editorial

**Published:** 1915-09

**Authors:** 


### EDITORIAL

The Atlanta Journal-Record of Medicine has its Business Office at 23 West Alabama Street where all business communications should be sent.

Remittances are payable to The Journal-Record of Medicine.

Concerning Original Communications and Editorial matters, address Richard R. Daly, M. D., 708 Empire Life Building, Atlanta, Ga.

Reprints of Original Articles will be furnished at cost price. Requests for them had best be made on the Manuscript.

Upon request, we will present, postpaid, to each contributor of an original article, twenty copies of The Journal-Record of Medicine containing such article.

### HEROISM IN THE LABORATORY.

If we had a feminine for the word we should use it because this time it is a woman who has distinguished herself by jeopardizing her health and life in the cause of the ministrations of science to humanity. Let all praise be hers as we add her name to the list of those who have been gifted with a far-seeing fancy and then led to the necessity for actual proof at the last moment of their investigations. Their clear vision stimulated them to the sacrifice when needed and the world has been benefitted beyond reckoning.

Her name is MARY DAVIES and she worked in the American Hospital at Nuilly, France as a nurse with DR. KENNETH TAYLOR who has been making laboratory and clinical studies of Gangrene for a long time. The war has made Gas Gangrene all too well known and the deaths from it have been about 100 per cent.

Taylor has made his preliminary report of his quinine remedy in the London Lancet for September 4 wherein he showed that his remedy has reduced the mortality in animals from 100 to 41 per cent. But there had been no opportunity to test it with the human host. It lacked just the proof it needed to secure for it a recognition and a trial by experimenters and clinicians.

Miss Davies who had been enthusiastic in her assistance realized this and without the knowledge or sanction of Taylor, she infected herself with virulent germs of gas gangrene which had been isolated in the laboratory and as soon as they begun their action, she sent for Taylor and told him what she had done. She knew that he never would have given his sanction to such an experiment, but it was evident that to prove the value of the drug, an unmixed infection should be ready for treatment.

Fortunately his work with her was successful and the disease was conquered. A great step toward the solution of that problem has been taken.

Dr. Taylor represents the University of Minnesota at the seat of war and has been studying the conditions as a teacher as well as an active clinician.

It is possible that there are those who will cavil at Miss Davies' act and say that she had no right to risk herself. They are of the kind that would save guinea pigs and sacrifice children; they call the doctor up nights and cheerfully expose him to any stresses and diseases they know of but they hate knowledge and they detest prevention.

---

### SOCIAL SERVICE TRAINING.

The need for assistance in philanthropic work along the line of follow-up help and also in special training for the care of tuberculosis and other so-called social diseases where the patients are long sufferers in the homes and where there is always danger of further spread of the disease, has long been felt by the physicians and others interested in the care of the indigent sick.

In addition it has been difficult to reach prosperous families at times where their need for advice and guidance was apparent but when the help might seem to be gratuitous or even intrusive.

This need has finally expressed itself in the establishment of The Social Training School of Georgia which has secured a charter for teaching properly qualified people both men and women, to do the social service that is necessary and showing them how to meet the problems that are constantly and increasingly demanding attention.

The Raoul Foundation for instance has field workers in Georgia striving to handle the tuberculosis problems so that the disease may be prevented and eventually eradicated. It is hampered in its work by the lack of properly trained nurses. It has positions for many additional workers which must be filled and in which it is the desire to install Southern people who have first-hand knowledge of the conditions to be met such as one from another section would take long in acquiring.

The School would give that training and it has already arranged for a competent Faculty to carry on the teaching. The curriculum will embrace all the major problems of Social Service and the teaching is to be by lectures and actual and practical study in the field.

The School is located at Atlanta and will enter upon its first term on the first of November, 1915.

The object is praiseworthy and tends to meet a necessity so aptly that we give it our earnest support and would call the attention of the Profession throughout the South to the opportunity it affords to graduate nurses and others with similar equipment to do post-graduate work that will be highly profitable to all concerned.

Information can be secured from Miss Rosa Lowe, Secretary, 23 East Cain Street, Atlanta, Ga.

---

### PELLAGRA.

A further study of the disease that is baffling us and enticing us at the same time is presented in this issue and coming from Dr. H. F. Harris, Secretary of the Georgia State Board of Health.

His plan contemplates the control of the disease through antibodies and predicates the origin of the toxin in the defective maise. He quotes extensively from other investigators in support of this idea.

It is his desire to secure as many patients as possible for laboratory study in conjunction with the family doctor and to do all in his power to discover the cause and cure of pellagra. His position and well-known ability entitle his request to serious consideration and it is in this spirit that we publish his crcular letter.

In the transactons of the Fulton County Medical Society as reported herein, there appears descriptions of cases of Sprue and one such case was exhibited. The differential diagnosis depended upon the lack of skin symptoms that have become classic in Pellagra, upon the course of the disease which lacks seasonal incidence and upon intestinal distension together with lack of gastric pain.

Despite these there seemed to be some doubt as to the identity of Sprue although one of the members who had lived in the Philippines said he had contracted the disease there and had recovered from his Sprue.

The conditions indicated by the allied diseases are of the greatest importance because it has come to pass in these days that we can say of the patients' to paraphrase St. Paul, behold they die daily.

---

### THE PREVENTIVE TREATMENT OF PELLAGRA BY INJECTIONS OF BAD MAIZE EXTRACTS.

To the Editor of Atlanta Journal-Record of Medicine,
Atlanta, Ga.:

While there can be no doubt that rest, good and assimilable food, and change of climate are of the utmost importance both in preventing recurrences of acute pellagrous attacks, and in warding off the graver symptoms in patients who have only suffered from the milder forms of the disease, and while such measures will in all probability continue to be our sheet-anchor in the treatment of such cases, much interest attaches to certain results which have been recently obtained in the prevention of the classical pellagrous onsets by means of injections of extracts of bad maize.

As is well known, it was long ago discovered by Devoto, and his assistant Ascoli, that pellagrins exhibit a hypersensibility to extracts of bad maize, and that the conclusions of these writers were fully confirmed by the later investigations of Volpino, Mariani, Bordoni, Alpago-Novello, Casa-Bianchi, Rondoni, and others. Somewhat later it occurred to Volpino that a state of resistance might be established by repeated injections of such extracts, and he, in conjunction with Bordoni, reported the results of his earlier investigations in the latter part of the year 1913. These observers began their work repeatedly injecting rabbits intravenously with extracts of bad maize. Ten days after discontinuing this treatment some of the blood serum of these animals was mixed with solutions of what they have called "pellagrogenina" of varying strengths, and the mixture administered subcutaneously to guinea pigs that had been sensitized to maize products, and it was found that the animals suffered no ill effects as a result of the injections, and it was, therefore, felt that antitoxic bodies were evidently contained in the serum used.

Following the foregoing experiments these investigators treated three patients in the fall of 1913 with injections of gradually increasing strength of extracts of bad maize with what appeared to be excellent results, and they have just reported a continuation of the work along these lines which was done in 1914, and make mention of the fact that the well-known pellagrologist Camurri has informed them by letter that he has also seen striking effects from the treatment.

Still more recently Finato and F. Novello have reported the results of the use of the extract in 14 cases, and have expressed themselves as having a high opinion of the efficacy of the treatment in many cases: in only 1 of the 14 persons treated was there even any doubt as to the beneficial effects secured.

Inasmuch as it takes some little time to carry out this plan of treatment, it is considered best for the patients to take it during the fall or winter.

In some instances the medicament has considered only of concentrated solutions of extracts of bad maize, and, what seem better, in other cases "pellagrogenina" has been employed. Whatever solution of bad maize is used, the utmost cae should be exerted in seeing that the solution is properly sterile; in some cases the investigators in testing the hypersensibility have sterilized the solutions by heat, but on the whole it would appear to be much better to filter the extracts through a Berkefeld or Chamberlin germ-proof filter, as only in this way would it be possible to preserve all of the component parts of the bad maize intact.

Gradually increasing strengths of the solutions are employed, the patient being carefully watched in order to avoid the occurrence of pronounced reactions, which, in case the solutions used are too strong, much resemble those obtained by tuberculin in patients with tuberculosis.

Ordinarily the injections are given every other day, and it requires about two months to complete the treatment, this being continued in gradually increasing doses until reactions no longer occur, even after the use of very strong solutions.

The results obtained by the well-known pellagrologists whose names have already appeared in this letter are such as to warrant a thorough investigation of this method of treatment. While it is certainly impossible ever to remove the extensive pathologic alterations that are always present in pellagrins, there is no question that we may by proper measures ward off the acuter manifestations in many instances, and should it be found that this treatment is of any decided value, it would certainly be a God-send to the unfortunate victims of the disease.

In order that the matter may be tested as early as possible, I have made preparations to treat a limited number of patients, free of charge, during the coming fall and winter, and will be glad to hear from physicians who feel interested. In order that this work may be of real scientific value, it is highly important that most thorough and complete histories of all patients should be obtained, and that a record be kept of the symptoms produced, particularly in the earlier stages, by the injections. For this reason I would greatly prefer that patients desiring this treatment should come to Atlanta and stay for at least a time in the earlier stages of the treatment, after which they may, if they prefer, go home and get their family physician to continue the injections. In cases where this is out of the question, and where the medical attendant will agree to furnish all of the data desired, with the subsequent results, I will be glad to furnish the extract, in properly sterilized, sealed glass tubes, free of all cost. Under such circumstances, however, it will be necessary for the medical attendant to write or wire the result of each injection, as the dose has to be gauged accordingly.

I would be very glad to hear from any physician who has patients upon whom he would like to try this treatment.

H. F. Harris

State Board of Health, State Capitol, Atlanta, Ga.

---

### MEETING GEORGIA SURGEON'S CLUB.

Rome, Ga., Sept. 11, 1915.

Dr. R. R. Daly, Editor,

Atlanta, Ga.,

Dear Doctor:

Will you announce in your columns that the Georgia Surgeons' Club will hold clinic days in Augusta, Wednesday, Feb. 16th. and Thursday 17th., 1915.

Please announce in another connection that there will be Surgical Clinics in Nashville, Tenn., Friday, Nov. 5th. and Memphis, Saturday, Nov. 6th, for those en route to the meeting of Southern Medical Association, Nov. 8-11. 1916, Dallas, Tex.

Yours truly,

R. M. Harbin, Secretary-Treasurer.

---

### SCOTTISH RITE ORTHOPEDIC HOSPITAL.

The Scottish Rite body of Masons have established an Orthopedic Hospital in Atlanta where crippled children of the South, whose parents are unable to pay may be treated without cost, for this is a free non-sectarian institution.

The Hospital has a present capacity of 20 beds and was opened September 23 with fifteen patients.

Dr. Michael Hoke is Surgeon and Chief of Staff, Dr. E. Victor Keller, Associate; Drs. O. L. Miller, Hugh Battey, assistants on Orthopedic sevice.; Dr. Theo Toepel, Physical Director; Drs. Floyd W. McRae, Frank Boland, General Surgeons; Drs. W. E. Campbell, Newton Craig, Eye, Ear, Nose and Throat Specialists: Drs. M. E. Turner, Chas. N. Hughes, S. L. Silverman, Dental Specialists,

Should any physician have a case he would care to have admitted, address application to

Mrs. W. C. Wardlaw,

143 Juniper St., Atlanta.

